# The variety of abomasal nematode communities of captive and free-roaming populations of European bison, *Bison bonasus* (L.): a morphometric and molecular approach

**DOI:** 10.1017/S003118202400088X

**Published:** 2024-09

**Authors:** Marta Gałązka, Katarzyna Filip-Hutsch, Daniel Klich, Wanda Olech, Krzysztof Anusz, Anna M. Pyziel

**Affiliations:** 1 Department of Food Hygiene and Public Health Protection, Institute of Veterinary Medicine, Warsaw University of Life Sciences (WULS), Nowoursynowska 159, 02-776 Warsaw, Poland; 2 Municipal Zoological Garden in Warsaw, Ratuszowa 1/3 03-461 Warsaw, Poland; 3 Department of Animal Genetics and Conservation, Institute of Animal Sciences, Warsaw University of Life Sciences – WULS, Ciszewskiego 8, 02-787 Warsaw, Poland

**Keywords:** *Bison bonasus*, enclosures, molecular biology, morphology, parasites, wildlife

## Abstract

Most studies concerning parasitic infections in European bison have been performed on free-ranging animals: comparatively little is known about the abomasal nematodes of captive wisents, which are widely used in reintroduction programmes. The aim of the study was to determine the infection level and species composition of abomasal nematodes in captive European bison in enclosures (including zoos) and breeding centres compared to free-ranging individuals. It also includes a morphological analysis of the parasites based on figures and measurement data. Altogether, 11 species of nematodes were detected, with both captive and free-ranging animals demonstrating similar species compositions. Among those, 2 species of blood-sucking nematodes were detected, including *Ashworthius sidemi* and *Haemonchus contortus*. Interestingly, *A. sidemi* was found in almost all free-roaming animals, but only in 1 captive European bison. In addition, *H. contortus* was predominant in captive animals. The morphological identification was confirmed molecularly for 5 nematode species: *A. sidemi*, *H. contortus*, *Ostertagia kolchida*, *O. ostertagi* and *Spiculopteragia boehmi*. The identification was performed using small subunit ribosomal rDNA. The study provides the first available set of specular lengths of the gastric nematodes of European bison, and the first molecular data of *O. kolchida* and *S. boehmi* derived from the same host species. Our findings may simplify the morphometrical and molecular identification of Trichostrongylidae species infecting European bison, and can be useful in developing new management strategies for populations of this near-threatened species in Europe.

## Graphical abstract

**Figure:**
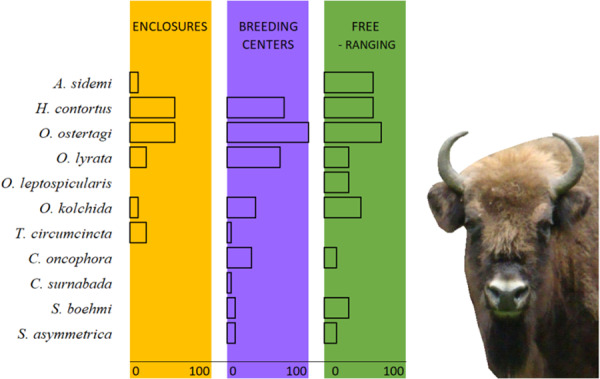

## Introduction

The global population of European bison (*Bison bonasus*) currently stands at over 10 000 individuals, with the largest groups inhabiting Poland, where the population was estimated at over 2300 free-living individuals and over 200 animals kept in captivity at the end of 2022 (Raczyński, 2023). However, the species remains near-threatened (ICUN), with its main threats being infectious diseases and parasite infestation (Kita and Anusz, 2006; Krasińska and Krasiński, 2013). European bison are known to host a wide diversity of parasites: studies have recorded 88 species, with many being typical for cattle, small ruminants and cervids, but also few species specific to European bison (Karbowiak *et al*., 2014*a*). Of these, the most numerous group in European bison are the nematodes, represented by 43 species, with gastrointestinal nematodes (GINs) being the most prevalent (Karbowiak *et al*., 2014*b*). Among the GINs, the blood-sucking nematodes of the Haemonchinae subfamily are considered to be of considerable pathogenic and economic importance in both domestic and wild ruminants worldwide (Osińska *et al*., 2010; Gilleard, 2013); 2 taxa of note include the alien species *Ashworthius sidemi*, brought to Poland with Asiatic deer, and *Haemonchus contortus*, which quickly develops resistance to anthelmintics used in its control (Gilleard, 2013; Demiaszkiewicz *et al*., 2017; Pyziel *et al*., 2018). Serious infestation can be fatal for the host, due to the blood loss from the mucosal lesions associated with parasite feeding causing haemorrhagic anaemia (Gilleard, 2013; Kołodziej-Sobocińska *et al*., 2016*a*, 2016*b*).

While the morphological aspects of the parasitofauna of free-ranging European bison are relatively well understood, little molecular data exists regarding their GINs. Also, little is known of the diversity of abomasal nematodes of captive wisents, as most studies of parasitic infections in European bison concern free-roaming individuals. In fact, these are the captive wisents that serve as a genetic reservoir for the species, and are more often translocated, i.e. between breeding centres, zoos or enclosures in Poland and abroad. Furthermore, while considerable data concerning the morphological identification of GINs is available for many ruminant species (Lichtenfels and Pilitt, 1991; Pyziel-Serafin *et al*., 2023), the morphology of the GIN flora of European bison remains sparse. A thorough understanding of the composition of the parasitofauna of captive animals will help limit the health risks associated with translocation of animals, prevent the spread of alien parasite species and monitor the efficacy of deworming of captive animals (Vadlejch *et al*., 2017; Gałązka *et al*., 2022, 2023).

The present study examines the infection level and composition of GINs in the abomasa of European bison, and compares the nematode species present in captive European bison with those in free-ranging animals. It also describes the morphometric characteristics of the identified nematodes to enable more accurate morphological diagnosis.

## Materials and methods

### Material collection and area of the study

Abomasa were collected during post-mortem examinations of 30 European bison, *viz.* 23 captive and 7 free-ranging individuals, in Poland during the years 2018–2023. All animals had died due to natural causes or culling for breeding and health reasons.

**Figure 1.**
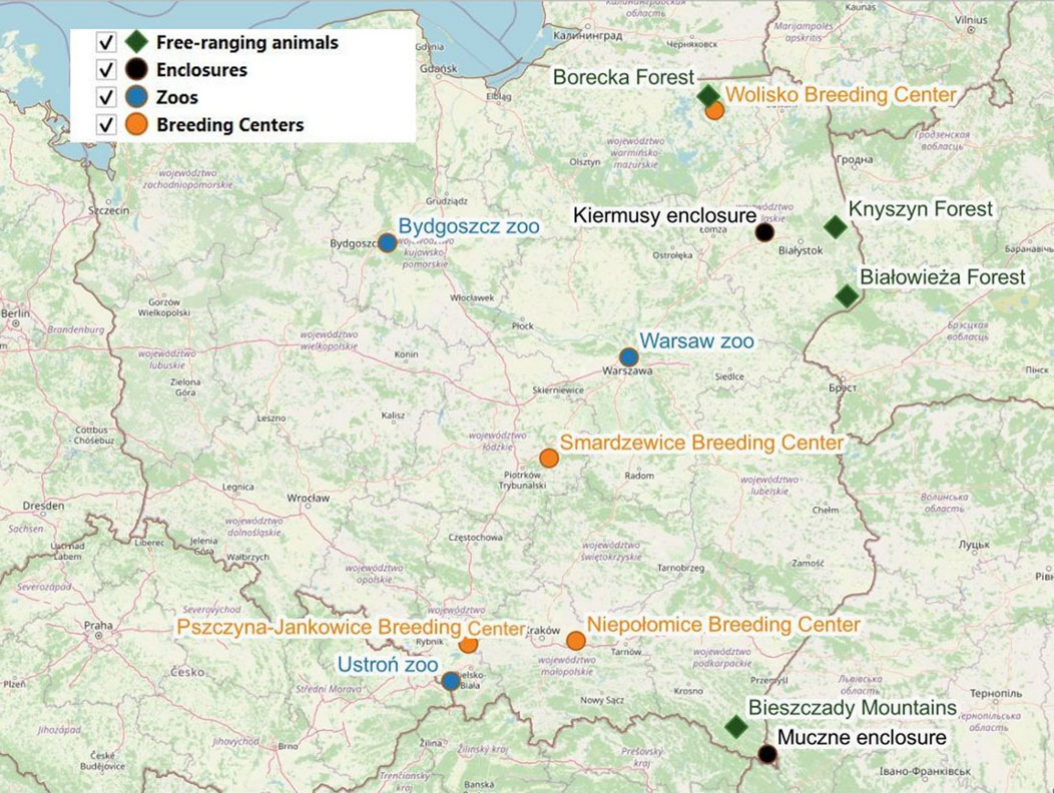
Location of examined European bison in Poland

The examined captive animals were kept in 4 breeding centres (Smardzewice Breeding Center, Niepołomice Breeding Center, Pszczyna-Jankowice Breeding Center and Wolisko Breeding Center), 3 zoos (Bydgoszcz zoo, Warsaw zoo and Ustroń zoo) and 2 enclosures (Kiermusy enclosure and Muczne enclosure) (Fig. 1). The free-ranging animals inhabited the Borecka Forest, Białowieska Forest, Knyszyńska Forest and Bieszczady Mountains (Fig. 1). Three management types were considered in the study: enclosures and zoos with smaller paddock area; breeding centres with larger pastures and free-living herds (Table 1). Detailed information about the number of animals examined in each location is available in the online Supplement (Tables S1, S2).

**Table 1.** Prevalence of abomasal nematodes of examined European bison according to management type

Location	Enclosures and zoos	Breeding centres	Free-ranging	Total
*n*	9	14	7	30
Nematode species	Prevalence (%)			
*A. sidemi*	11.1		57.1	16.7
*H. contortus*	55.6	71.4	57.1	63.3
*O. ostertagi*	55.6	100	71.4	80.0
*O. lyrata*	22.2	64.2	28.6	43.3
*O. leptospicularis*			28.6	6.7
*O. kolchida*	11.1	35.7	42.9	30.0
*T. circumcincta*	22.2	7.1		10.0
*C. oncophora*		28.6	14.3	16.7
*C. surnabada*		7.1		3.3
*S. boehmi*		14.3	28.6	13.3
*S. asymmetrica*		14.3	14.3	10.0

*n*, number of examined animals.

Parasitological dissections were performed in the field, according to the principles of dissecting technique and parasitological procedures (Dróżdż, 1966; Malicka, 2008). In each case, the abomasa were removed from the abdominal cavity, ligated at both ends and secured for examination. The material was transported to the laboratory and examined immediately, or frozen at −20°C and examined after thawing.

Each abomasum was placed in standing water. Its wall was cut along the greater curvature and the contents were then decantated and a sample of 1/10 of the total volume of the sediment was prepared. The sediment was poured into Petri dishes (in portions) and investigated under a Delta Optical SZ-450 T stereomicroscope (Delta Optical, Mińsk Mazowiecki, Poland) using 40 × magnification. The observed GINs were isolated with a use of a dissecting needle and counted. Afterwards, the male and female individuals were separated and each group was preserved in 70% ethanol.

### Morphological identification of nematodes

Male nematodes were isolated from 1/10 of the abomasal contents. These were used for morphological species identification due to the species-specific structure of the copulatory bursa. The caudal part of the body was cut off with scalpel and placed in Amman's lactophenol (10 g of phenol + 10 g of lactic acid + 20 g of glycerol + 10 g of distilled water) for 20 min to increase the translucency of the cuticle and improve the visibility of the spicules. The rest of the body was placed in a labelled vial filled with 70% ethanol for subsequent species verification using molecular biology techniques. When removed from lactophenol, the caudal body parts of male nematodes were placed on a basal slide and identified to the species level; identification was based on the shape and length of the spicules and copulatory sac under a LAB40 microscope (OPTA-TECH, Warsaw, Poland) under 100 × to 400 × magnification (Dróżdż, 1966, 1995; Dróżdż *et al*., 1998). The spicules were photographed and measured with a digital camera and OPTA View-15 2019 software (OPTA-TECH). The female nematodes were identified to the subfamily level and counted.

### Molecular identification of nematodes

Genomic DNA was extracted individually from the ethanol-preserved anterior male parts of *A. sidemi*, *Cooperia oncophora*, *H. contortus*, *O. leptospicularis*, *O. kolchida* (a minor morph of *O. leptospicularis*), *O. ostertagi*, *O. lyrata* (a minor morph of *O. ostertagi*), *Spiculopteragia boehmi* and *Teladorsagia circumcincta*. The extraction was performed using a NucleoSpic Tissue DNA extraction kit (Macherey-Nagel, Düren, Germany) according to the manufacturer's protocol.

Identification was performed by PCR using various combinations of primer sets. A partial region of the internal transcribe spacer 2 (ITS-2) and large subunit (LSU) of the ribosomal DNA was amplified using the following set of primers: forward-NC1 (5′-ACG TCT GGT TCA GGG TTG TT-3′) and reverse-NC2 (5′-TTA GTT TCT TTT CCT CCG CT-3′) according to Gasser *et al*. (1993). Additionally, 2 sets of primers amplifying a partial region of the small subunit ribosomal rDNA (SSU) were designed for the purpose of this study, namely: forward-N380F (5′-AAG CGA GCA GGC GCG AAA C-3′) and reverse-N1690R (5′-ACC CGG TTC AAG CCA TTG CGA-3′); forward-N350F (5′-GAG CCT TAG AAA CGG CTA CCA CAT CCA-3′) and reverse-N1287R (5′-AGC AGG CTA GAG TCT CGC TCG T-3′).

Other combinations of primer sets targeting SSU were also used, including: forward SSU07 (5′-AAA GAT TAA GCC ATG CAT G-3′) and reverse BNR1 (5′-ACC TAC AGA TAC CTT GTT ACG AC-3′); forward SSU07 and reverse N1070R (5′-TTG CAA CCA TAC TAC CCC AGG AAC CGA A-3′); forward N800F (5′-GGG CAT TCG TAT CCC TGC GCG AGA G-3′) and reverse NBR1; forward NF50 (5′-TGA AAC TGC GAA CGG CTC AT-3′) and reverse BNR.

All PCRs were performed in a T100 thermal cycler (Bio-Rad, Hercules, CA, USA) in a volume of 50 *μ*L. Each 50 *μ*L PCR reaction contained 20 *μ*L of Molecular Biology Reagent Water (Sigma-Aldrich, St. Louis, MO, USA), 25 *μ*L of AccuStart II PCR ToughMix (×2 concentation) (Quantabio, Beverly, MA, USA), 1 *μ*L of GelTracl Loading Dye (×50 concentration) (Quantabio), 1 *μ*L of forward primer (20 mm), 1 *μ*L of reverse primer (20 mm), and 2 *μ*L of template DNA. The conditions for PCRs were as follows: 94°C for 2 min to denature the DNA, 35 cycles at 94°C for 45 s, 60°C (for ITS-2 and LSU)/57°C (for SSU) for 60 s, and 72°C for 45 s, and a final extension of 10 min at 72°C to ensure complete amplification.

The PCR products were verified on 1% agarose gel containing ethidium bromide (0.5 *μ*g mL^−1^), with Gene Ruler 100 bp DNA Ladder (Thermo Fisher Scientific, Waltham, MA, USA) loaded as a reference. The products were identified by exposure to UV light using a Gel Doc XR+ (Bio-Rad) gel documentation system, equipped with Image Lab 6.1 Software (Bio-Rad Laboratories, USA). The PCR products were purified using the NucleoSpin Gel and PCR Clean-up kit (Macherey-Nagel), eluted with 30 *μ*L of Molecular Biology Reagent Water (Sigma-Aldrich) and sequenced in both directions by Genomed S.A. (Warsaw, Poland) using the primers previously used for amplification (5 mm). The sequences were assembled into contigs using CodonCode Aligner version 8.0 (CodonCode Corporation, Centerville, MA, USA). The obtained nucleotide sequences were compared to the NCBI database of sequences using the basic local alignment search tool (BLAST) (http://www.ncbi.nlm.nih.gov/BLAST/) and submitted to the GenBank database.

### Statistical analysis

Due to the low intensity of nematode infection in the examined European bison, statistical analysis allowing for the identification of factors explaining the intensity of nematode infection was limited. Therefore, the analysis was restricted to 2 areas. The first evaluated the number of nematode species according to European bison maintenance type (enclosures (including zoos), breeding centres and free-living herds) using the Kruskall-Wallis test with Dunn's post-hoc test with Bonferroni correction.

In addition, the number of nematodes of each selected nematode species was analysed. Briefly, after analysing the number of nematodes present, 3 species were subjected for detailed statistical analysis: *H. contortus, O. ostertagi* and *O. lyrata*. The numbers of these species were assessed for collinearity using Pearson's r and Kendall's b correlation coefficients. Following this, the number of nematodes in each species was analysed separately based on a generalized linear model with a negative binomial distribution and a log link function. The number of nematodes in the European bison was used as the dependent variable, while bison sex, maintenance type [enclosures (including zoos), breeding centres and free-living herds], and northern gradient were used as explanatory variables. The northern gradient variable was introduced into the analysis to account for the effect of the north-south gradient on the occurrence of various parasite species (e.g. Thieltges *et al*., 2011; Klich *et al*., 2022) and the distribution of samples from the north to the south of Poland (Fig. 1). Each model underwent a selection process comparing all model variants (variable composition, including a null model), and the highest ranked model was selected based on the Akaike information criterion, i.e. the highest AIC value (Burnham and Anderson, 2002). All statistical analyses were performed with IBM SPSS v29.0 (Armonk, New York).

## Results

### Morphological identification of nematodes

**Figure 2.**
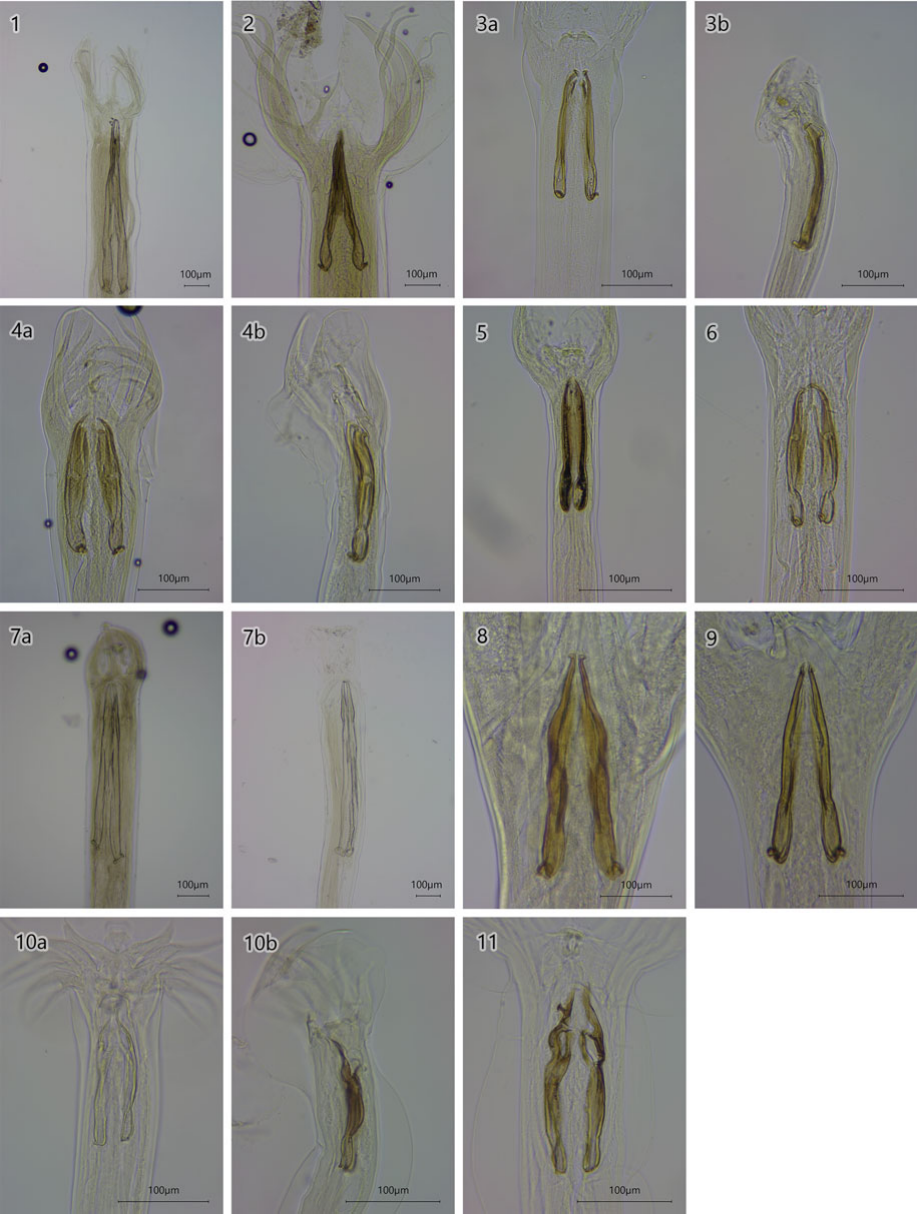
Male caudal body parts of abomasal nematodes found in European bison: 1 – *Ashworthius sidemi* (×10); 2 – *Haemonchus contortus* (×10); 3 a, b – *Ostertagia ostertagi* (×20); 4 a, b– *Ostertagia lyrata* (×20); 5 – *Ostertagia leptospicularis* (×20); 6 – *Ostertagia kolchida* (×20); 7 a, b– *Teladorsagia circumcincta* (×10); 8 – *Cooperia oncophora* (×20); 9 – *Cooperia surnabada* (×20); 10 a, b – *Spiculopteragia boehmi* (×20); 11 – *Spiculopteragia asymmetrica* (×20).

In the study, 11 GIN species were identified: *H. contortus*, *A. sidemi*, *O. ostertagi*, *O. lyrata* (a minor morph of *O. ostertagi*), *O. leptocpicularis*, *O. kolchida* (a minor morph of *O. leptospicularis*), *T. circumcinta*, *C. oncophora*, *C. surnabada*, *S. boehmi* and *S. asymmetrica* (Fig. 2). Among these, *H. contortus, A. sidemi, O. ostertagi, O. lyrata, O. kolchida, C. oncophora, S. boehmi* and *S. asymmetrica* were noted in both free-roaming and captive animals. However, *T. circumcincta* and *C. surnabada* were exclusively found in captive bison and *O. leptospicularis* in free-roaming animals. The overall prevalence of GIN infection in examined European bison was 90% (i.e. 27 positive out of 30 examined abomasa). The median and mean intensity of GIN infection were respectively 725 and 5065.7 per animal (range 0–47 030 nematodes per animal). The general prevalence was the highest in breeding centres (100%), followed by free-ranging herds (85.7%) and enclosures including zoos (77.8%).

The most common, and also most abundant, species was *Ostertagia ostertagi* (prevalence: 80%), followed by *H. contortus*, *O. lyrata* and *O. kolchida* (prevalence: 63.3, 43.3 and 30%, respectively) (Table 1). The rarest nematode species was *C. surnabada* (prevalence: 3.3%) (Table 1). Regarding maintenance type, *O. ostertagi* and *H. contortus* were found to have similar prevalences in free-roaming and captive European bison (both types of maintenance), whereas the greatest difference between maintenance types was noted for *A. sidemi*: the second most prevalent species in free-ranging animals (prevalence: 57.1%) but one of the rarest in captive wisents (prevalence in enclosures and zoos 11.1%, breeding centres 0%) (Table 1).

GIN composition was found to be related to the management type of the European bison. *Ashworthius sidemi* occurred in most free-ranging herds, but was noted in only one enclosure, where the species was also present in the wild population (Table 1, online Supplementary Table S1). *Teladorsagia circumcincta* and *C. surnabada* were only found in captive animals; *O. leptospicularis* was noted only in free-ranging animals; all other GINs were present in both captive and free-ranging animals. In addition, mixed infections comprising 2 to 8 GIN species were noted in 25 cases; however, no relationship was found between the number of species and the type of maintenance.

**Figure 3.**
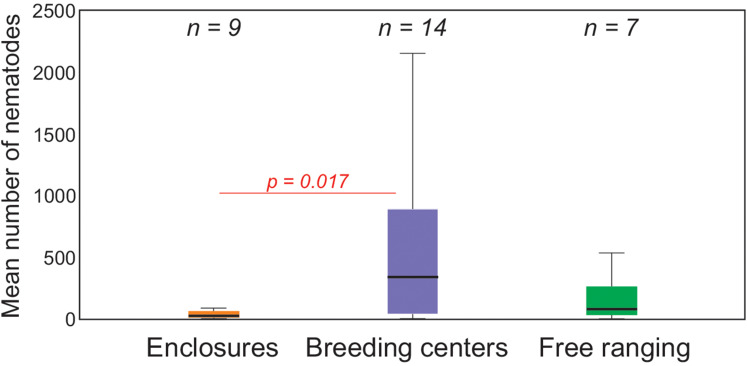
Boxplot of intensity of infection with GINs in European bison with regard to maintenance type. In Kruskall–Wallis test only animals in breeding centres presented higher intensity of infection with GINs than in enclosures and zoos (enclosures) (Dunn's test statistic = 10.38, *P* = 0.017). Free ranging animals didn't differ significantly from those in enclosures and zoos (Dunn's test statistic = 5.67, *P* = 0.604) nor from breeding centres (Dunn's test statistic = 4.71, *P* = 0.741, for Kruskal–Wallis test: *P* = 0.022) (*n*, number of examined animals).

The parasite loads differed regarding the maintenance type. The European bison in breeding centres presented higher intensity of infection with GINs than in enclosures and zoos (Fig. 3). Free ranging animals did not differ significantly from those in enclosures and zoos. Regarding the most prevalent GINs, the highest average and median values, as well as the widest range of specimens, were observed for *O. ostertagi* in breeding centres (351.1; 87.5; 10–21 230, respectively). In free-ranging European bison the highest intensity of infection was noted also for *O. ostertagi* (40–800), with the highest average and median (25.6 and 5, respectively) (Table 2).

**Table 2.** Intensity of abomasal nematodes of examined European bison according to management type

Location	Enclosures and zoos			Breeding centres			Free-ranging		
*n*	9			14			7		
Intensity									
Nematode species	Average	Median	Range	Average	Median	Range	Average	Median	Range
*A. sidemi*	20^a^						12.6	4	40–710
*H. contortus*	5.9	5	50–230	16.3	3	10–580	20.3	5	50–680
*O. ostertagi*	14.9	1	10–610	351.1	87.5	10–21 230	25.6	5	40–800
*O. lyrata*	0.7	0	10–680	14.6	6.5	10–680	0.3	0	10
*O. leptospicularis*							0.7	0	20–30
*O. kolchida*	40^a^			2.5	0	10–310	3	0	10–160
*T. circumcincta*	0.6	0	10–40	20^a^					
*C. oncophora*				1.2	0	10–50	40^a^		
*C. surnabada*				40^a^					
*S. boehmi*				0.36	0	10–40	2.4	0	70–100
*S. asymmetrica*				1.1	0	80	20^a^		

*n*, number of examined animals.

a Nematodes found only in 1 examined European bison.

**Figure 4.**
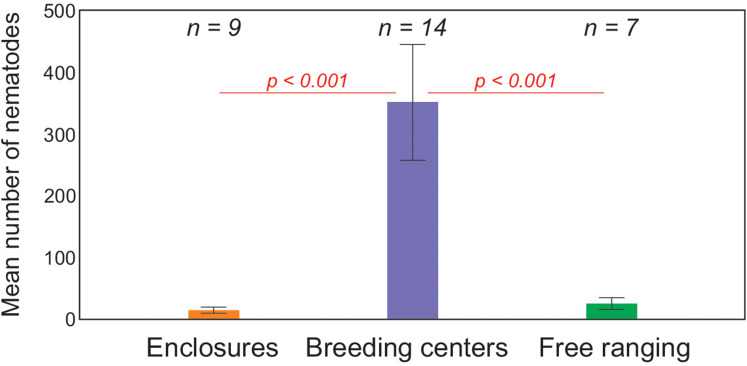
Mean (±s.e.) number of *O. ostertagi* specimens in European bison with regard to maintenance type. Animals in breeding centres differed significantly from those in enclosures and free-ranging animals (*P* < 0.001 in both cases) in generalized linear model. Free ranging animals did not differ significantly from animals in enclosures (*P* = 0.336) (for whole model: *χ*^2^ = 50.81, *P* < 0.001) (*n*, number of examined animals).

**Figure 5.**
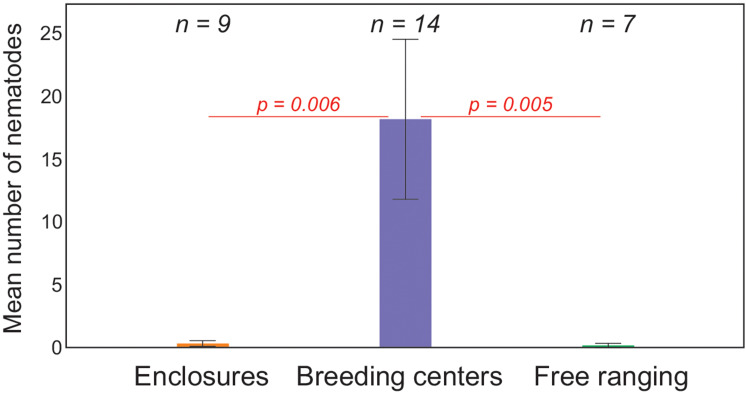
Mean (±s.e.) number of *O. lyrata* specimens in European bison with regard to maintenance type. Animals in breeding centres differed significantly from those in enclosures and free-ranging animals (*P* < 0.01 in both cases) in generalized linear model. Free ranging animals did not differ significantly from animals in enclosures (*P* = 0.529) (for whole model: *χ*^2^ = 54.18, *P* < 0.001) (*n*, number of examined animals).

**Figure 6.**
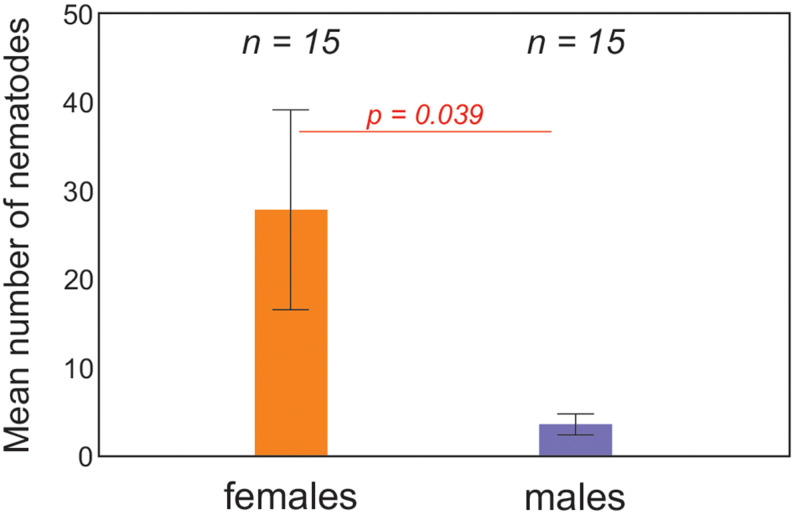
Mean (±s.e.) number of *H. contortus* specimens in European bison with regard to sex of animals. Females differed significantly from males (*P* = 0.039) in generalized linear model (for whole model: *χ*^2^ = 26.51, *P* < 0.001) (*n*, number of examined animals).

*Ostertagia ostertagi* and *O. lyrata* were found at significantly higher intensities in animals kept in breeding centres than in enclosures or in wild herds (Figs 4 and 5), but such a difference was not observed in *H. contortus* (online Supplementary Table S3). Free ranging animals did not differ significantly from animals in enclosures for all 3 nematodes species. *Haemonchus contortus* was present at significantly higher intensity in cows than in bulls (Fig. 6), but for *O. ostertagi and O. lyrata* no sex differences were found (online Supplementary Table S3). Interestingly, the intensity of *H. contortus* infection decreased as the location became more northerly, regardless of maintenance type; for *O. ostertagi and O. lyrata* northern gradient was not significant in the model (online Supplementary Table S3). More detailed data about the prevalence and intensity of parasitic infections in the examined European bison in all locations is given in the online Supplementary material (Tables S1 and S2).

Nematode species were identified based on the morphology and length of the male copulatory bursa and the spicules; the morphological differences between *H. contortus* and *A. sidemi* can be seen in Fig. 2. In *H. contortus*, the lateral rib is clearly visible in the spicules. This difference allows accurate identification of this species by light microscopy, as confirmed by our present molecular analysis (Fig. 2.2). However, all species demonstrated a wide range of spicule lengths. In *A. sidemi*, *T. circumcinta* and *S. boehmi*, the median spicule length was significantly higher than the mean length due to the occurrence of significantly lower values (Table 3).

**Table 3.** Comparison of the spicule length of abomasal nematodes of European bison

Species	*n*	Average ± s.d.	Median	Range [um]
*Ashworthius sidemii*	6	686.4 ± 144.56	699.5	(436–877)
*Haemonchus contortus*	22	416.6 ± 21.52	414	(370–475)
*Ostertagia ostertagi*	60	213.5 ± 9.88	214	(170–235)
*O. lyrata*	43	216.1 ± 12.91	217	(178–245)
*O. leptospicularis*	4	168.4 ± 5.13	168.5	(160–176)
*O. kolchida*	4	195.9 ± 23.27	189.5	(169–234)
*Teladorsagia circumcincta*	7	583.7 ± 151.62	694	(351–718)
*Cooperia oncophora*	10	266.6 ± 8.25	266	(254–285)
*C. surnabada*	3	241.3 ± 12.45	242.5	(226–255)
*Spiculopteragia boehmi*	8	154.7 ± 22.01	161.5	(99–175)
*S. asymmetrica*	7	254.3 ± 32.33	252	(216–303)

s.d., standard deviation.

### Molecular identification of nematodes

Nucleotide sequences were obtained for 5 species of abomasal nematodes, including *A. sidemi, H. contortus, O. ostertagi, O. kolchida* and *S. boehmi* (Table 4). None of the primer sets used in the investigation provided a PCR product for all nematode species included in the study. The following primer pars proved to be completely unsuccessful for the purpose of this research: SSU07 + BNR1, SSU07 + N1070R, N800F + NBR1 and NF50 + BNR1. The reason for this is the fact that no PCR product was produced by these sets of primers. However, the remaining primer sets were selectively useful, i.e. they could accurately identify only certain nematode species.

**Table 4.** Obtained nucleotide sequences of abomasal nematodes of captive European bison

Organism	Gene	Primer F	Primer R	GenBank no.	Length (bp)
*Ashworthius sidemi*	ITS2 + LSU	NC1	NC2	OP787564.1	239
	SSU	N380F	N1690R	OP320470.1	1132
*Haemonchus contortus*	ITS2 + LSU	NC1	NC2	OP783972.1	255
	SSU	N380F	N1690R	OP577478.1	977
	SSU	N380F	N1690R	OP320502.1	1191
*Ostertagia kolchida*	SSU	N350F	N1287R	OP320518.1	839
*Ostertagia ostertagi*	ITS2 + LSU	NC1	NC2	OP787667.1	257
*Spiculopteragia boehmi*	SSU	N380	N1690R	OP320506.1	1211

*NC1: 5′-ACGTCTGGTTCAGGGTTGTT-3′; NC2: 5′-TTAGTTTCTTTTCCTCCGCT-3′; N380F: 5′-AAGCGAGCAGGCGCGAAAC-3′; N1690R: 5′-ACCCGGTTCAAGCCATTGCGA-3′; N350F: 5′-GAGCCTTAGAAACGGCTACCACATCCA-3′; N1287R: 5′-AGCAGGCTAGAGTCTCGCTCGT-3′

The general primer set NC1 + NC2 obtained sequences for ITS2 and LSU from *A. sidemi*, *H. contortus* and *O. ostertagi*; the products varied in length from 239 to 257 bp. Partial SSU sequences were obtained for *A. sidemi*, *H. contortus*, *O. kolchida* and *S. boehmi*; these varied in length from 830 bp (for *O. kolchida*) to 1211 bp (for *S. boehmi*). The 350F + N1287R set was useful for only *O. kolchida*, whereas primer set 380F + N1690R obtained sequences from the 3 remaining nematode species.

## Discussion

In total, 11 GIN species of the family Trichostrongylidae were isolated from the alimentary tracts of European bison. Although our study concerned mostly captive animals, the GIN species composition was similar to that of free-living European bison obtained by other authors worldwide (Dróżdż *et al*., 1994; Karbowiak *et al*., 2014*a*, 2014*b*; Demiaszkiewicz *et al*., 2020). GINs are the most significant parasites of European bison and other grazing ruminants (Karbowiak *et al*., 2014*b*) and the general prevalence of parasitic infections in our research was high, reaching 90%.

Our morphometric analysis found that most GIN species demonstrated high size ranges, and this corresponded with values obtained in other ruminant species (Lichtenfels and Pilitt, 1991; Jacquiet *et al*., 1997; Pyziel-Serafin *et al*., 2023). The morphological identification was made possible by the structure of the nematode spicules, and these results were confirmed in the molecular analysis. Our results represent the first available set of specular lengths of GINs in European bison. Nevertheless, due to the limited sample size the descriptions may not be comprehensive for all possible sizes and it is advised to perform further measurements of nematode spicules.

Unfortunately, limited data is available on the nucleotide sequences of abomasal nematodes of ruminants, and as it was not possible to acquire many of the sequences of the SSU and ITS genetic markers in the studied GIN from European bison. Even so, a nucleotide sequence was acquired from *O. kolchida* (GenBank: OP320518.1), and this is the first to be made available in GenBank regarding any genetic marker gene of the species. Similarly, the obtained SSU sequence of *S. boehmi* (GenBank: OP320506.1) is the first SSU sequence of the species available, and first molecular data from *S. boehmi* isolated from European bison. In addition, the acquired second sequence of ITS2 and LSU of *O. ostertagi* (GenBank: OP787667.1) is the second to be derived from European bison. The previous molecular data on the species were obtained from Lowland-Caucasian wisent from Avesta Visentpark in Sweden (GenBank: KX358862.1) (Pyziel *et al*., 2018).

In contrast, sequences of blood-sucking *H. contortus* and *A. sidemi* are more available. In addition to the data on *H. contortus* obtained in this study (GenBank: OP577478.1, OP320502.1), sequences have been derived from European bison inhabiting Almindingen in Danish island, Bornholm (GenBank: ON677956.1, ON677957.1, ON677958.1) and Avesta Visentpark in Sweden (GenBank: KX358860.1). Our data regarding the molecular features of an alien *A. sidemi* isolated from European bison are the third set to be published (GenBank: OP787564.1, OP320470.1). This issue was discussed previously by Moskwa *et al*. (2014) in Poland (GenBank: KF414629.1) and by Vadlejch *et al.* (2017) in the Czech Republic (GenBank: KX228148.1, KX228149.1).

The most prevalent GIN was *O. ostertagi*, a parasite typical for bovids and considered one of the most common GINs of European bison. It has been observed in both captive and free-living animals since the beginning of the European bison restitution (Karbowiak *et al*., 2014*b*). Other parasites typical of bovines, like *H. contortus* and *O. lyrata* were also very common, followed by *O. kolchida*, characteristic for cervids. Indeed, during its reintroduction, the European bison has become a host for many parasite species originally characteristic for other mammals (Dróżdż *et al*., 1998, 2002), which is consistent with our study.

As the development and spread of parasitic infections are affected by many environmental and individual factors (Kołodziej-Sobocińska, 2019), some differences between captive and free-living animals might be expected. In our study, 2 nematode species, *T. circumcincta* and *C. surnabada*, were isolated only from European bison in captivity. Both nematodes are considered typical parasites of domestic ruminants and have been rarely reported in free-living animals (Demiaszkiewicz and Pyziel, 2010; Karbowiak *et al*., 2014*b*). In contrast, *O. leptospicularis* was not present in captive animals but isolated only from free-living ones. *Ostertagia leptospicularis* is primarily a parasite of cervids (Wyrobisz-Papiewska *et al*., 2021), but it has been adapted by free-living European bison as a result of inter-species parasite transmission (Dróżdż *et al*., 1989; Karbowiak *et al*., 2014*b*). Previous studies indicate that the parasitofauna of free-ranging bison might be enriched with species typical for cervids which are rarely present in captive individuals (Dróżdż *et al*., 2002).

Similar differences between captive and free-ranging European bison were observed in relation to nematodes from Haemonchiinae subfamily, including *H. contortus* and *A. sidemi*. *Ashworthius sidemi* was imported to Poland with sika deer (*Cervus nippon*) from Asia in the end of XXth century. It has since become a common parasite of free-living European bison (Dróżdż *et al*., 1998; Demiaszkiewicz *et al*., 2009; Kołodziej-Sobocińska *et al*., 2016*a*, 2016*b*); however, in the present study, it was isolated only from one individual in enclosure, and the predominant Haemonchinae species in most captive European bison was *H. contortus*. Other authors attribute the presence of *A. sidemi* in captive European bison to accidental contact with infected wild ruminants in the study area, as enclosure is not always a sufficient barrier to transmission of infectious diseases (Kowal *et al*., 2012; Rendón-Franco *et al*., 2013; Mazur *et al*., 2018).

Overall, the median and mean intensity of infection (725; 5065.7 nematodes per animal, respectively) were lower than those reported in previous studies of European bison in Poland (Dróżdż *et al*., 1989; Demiaszkiewicz *et al*., 2018; Kołodziej-Sobocińska *et al*., 2018). The lower parasite burden may be related to management type, e.g. captive animals are dewormed biannually to prevent the spread of parasitic disease (Viggers *et al*., 1993), while the free-ranging European bison are not receiving anthelmintics due to lack of control over the drug intake and a potential development of anthelmintic resistance. Nonetheless, the infection intensity was higher in animals in breeding centres compared with animals in enclosures and zoos and free-living herds; this may be due to the other factors faced by captive animals, such as higher population density, close proximity to other animals (Gałązka *et al*., 2023), higher stress and specific environmental conditions (Papini *et al*., 2012). Moreover, herds living in breeding centres were larger than those enclosures and zoos, which may pose a potential difficulty in estimating the body mass of all animals and administering a correct dosage of anthelmintic drugs to a group, which may lead to inefficient deworming and development of drug resistance in parasites (Gałązka *et al*., 2023). However, only a relatively small number of samples, especially from free-living individuals were examined and further study with emphasis on management type in European bison should be undertaken.

Among the examined European bison, a higher infection intensity of *H. contortus* was found among females than males; however, this pattern was not noted for other GINs. Female-biased parasitism has been reported in European bison before (Krasińska *et al*., 2000; Pyziel *et al*., 2011; Kołodziej-Sobocińska, 2019; Filip-Hutsch *et al*., 2022). It has been attributed to the higher exposure of females to parasites due to their living among other animals, which favours transmission of diseases between individuals. Their susceptibility to parasitic infection may also be enhanced by immunosuppression caused by pregnancy and lactation (Lloyd, 1983).

For one of the most common GINs, *H. contortus*, infection intensity was found to change depending on the latitude. While other authors also report a tendency for increasing parasite species richness when moving away from the equator, there is no strong evidence indicating that latitude has a direct effect on mammal parasitofauna (Kamiya *et al*., 2014). It is more likely that affect the latitudinal patterns of helminth diversity are influenced indirectly by several additional factors, such as temperature, precipitation and host availability (Villalobos-Segura *et al*., 2020). Moreover, most sheep and goat farming occurs in the south of Poland, while more cattle farming occurs in the north, which might be taken under consideration in explanation of the higher intensity of infection with nematodes commonly associated with small ruminants (*H. contortus*) in the southern enclosure locations (Łączyński *et al*., 2022). However, this issue is more complex, as indicated by regional differences in the observed contacts between European bison and cattle which were not always corresponding to cattle numbers in given region (Klich *et al*., 2023). Due to limited access to invasive sampling from endangered species the sampling was not randomized, and since further research ought to be conducted to investigate potential correlations, the sampling protocol requires to consider this methodological aspect in the future.

As captive-bred animals are widely used in reintroduction programmes (Mathews *et al*., 2006; Kołodziej-Sobocińska *et al*., 2018), it is especially important to monitor their health status. Despite this, few studies have examined the extent and nature of parasitic infections in captive European bison. Our study has implications for managing the captive European bison reintroduction process. Although individuals from breeding centres are better adapted to natural conditions, they may constitute a significant reservoir of nematodes. However, the intensity of GIN infection is not high in all individuals, so a preliminary assessment of this intensity should be carried out before selecting specimens for transportation. Moreover, animals in breeding centres may be reservoirs of nematode species that occur in the local environment, even though they do not live in the wild. Therefore, if a species of nematode unknown to the target place for reintroduction was found in the environment in the source region a thorough analysis of the nematode species composition of animals from breeding centre before transportation is highly justified. This study provides new data about GINs in captive European bison in Poland, which might simplify the morphometrical and molecular identification of Trichostrongylidae species, and help develop new management strategies for the European bison population, such as, preventing from transporting alien or potentially threatening species to new locations and free-living populations.

## Supporting information

Gałązka et al. supplementary material Gałązka et al. supplementary material

## Data Availability

The data that support the findings of this study are available from the corresponding author (MG).

## Supplementary material

The supplementary material for this article can be found at https://doi.org/10.1017/S003118202400088X
