# Supplementary material for: The variety of abomasal nematode communities of captive and free-roaming populations of European bison, Bison bonasus (L.): a morphometric and molecular approach
Source: Parasitology. 2024 Nov 11;151(11):1175–84. doi: 10.1017/S003118202400088X (PMC11894010; doi:10.1017/S003118202400088X)
Supplement: Gałązka et al. supplementary material [file S003118202400088Xsup001.docx]

Table S1. Prevalence of abomasal nematodes of examined European bison according to location of the host

| Location | n | *A. sidemi* | *H. contortus* | *O. ostertagi* | *O. lyrata* | *O. leptospicularis* | *O. kolchida* | *T. circumcincta* | *C. oncophora* | *C. surnabada* | *S. boehmi* | *S. asymmetrica* |
| --- | --- | --- | --- | --- | --- | --- | --- | --- | --- | --- | --- | --- |
|  |  | Prevalence (%) | | | | | | | | | | |
| Enclosures and zoos | 9 |  |  |  |  |  |  |  |  |  |  |  |
| Muczne | 1 | 100* |  | 100* |  |  | 100* |  |  |  |  |  |
| Kiermusy | 3 |  | 100 | 66.7 | 33.3 |  |  |  |  |  |  |  |
| Bydgoszcz ZOO | 1 |  |  | 100* | 100* |  |  |  |  |  |  |  |
| Ustroń ZOO | 2 |  | 100 | 50 |  |  |  | 100 |  |  |  |  |
| Warsaw ZOO | 2 |  |  |  |  |  |  |  |  |  |  |  |
| Breeding centers | 14 |  |  |  |  |  |  |  |  |  |  |  |
| Pszczyna-Jankowice | 2 |  | 100 | 100 | 100 |  | 50 |  | 50 | 50 | 100 | 50 |
| Niepołomice | 3 |  | 100 | 100 | 66.7 |  |  |  | 66.7 |  |  | 33.3 |
| Smardzewice | 6 |  | 50 | 100 | 33.3 |  | 33.3 |  | 16.7 |  |  |  |
| Wolisko | 3 |  | 66.7 | 100 | 100 |  | 66.7 | 33.3 |  |  |  |  |
| Free-ranging | 7 |  |  |  |  |  |  |  |  |  |  |  |
| Białowieża Forest | 2 | 100 | 50 | 100 | 100 | 100 | 50 |  | 50 |  | 50 |  |
| Bieszczady Mountains | 3 | 33.3 | 66.7 | 100 |  |  | 66.7 |  |  |  | 33.3 | 33.3 |
| Borecka Forest | 1 |  |  |  |  |  |  |  |  |  |  |  |
| Knyszyn Forest | 1 | 100* | 100* |  |  |  |  |  |  |  |  |  |
| Total | 30 | 16.7 | 63.3 | 80.0 | 43.3 | 6.7 | 30.0 | 10.0 | 16.7 | 3.3 | 13.3 | 10.0 |
| Captive |  | 4.3 | 65.2 | 82.6 | 47.8 |  | 21.7 | 13.0 | 17.4 | 4.3 | 8.7 | 8.7 |
| Free-ranging |  | 57.1 | 57.1 | 71.4 | 28.6 | 28.6 | 42.9 |  | 14.3 |  | 28.6 | 14.3 |

*Only one examined European bison

Table S2. Intensity of abomasal nematodes of examined European bison according to location of the host

| Location | n | *A. sidemi* | *H. contortus* | *O. ostertagi* | *O. lyrata* | *O. leptospicularis* | *O. kolchida* | *T. circumcincta* | *C. oncophora* | *C. surnabada* | *S. boehmi* | *S. asymmetrica* |
| --- | --- | --- | --- | --- | --- | --- | --- | --- | --- | --- | --- | --- |
|  |  | Intensity | | | | | | | | | | |
|  |  | Average; Median; (Range) | | | | | | | | | | |
| Enclosures and zoos | 9 |  |  |  |  |  |  |  |  |  |  |  |
| Muczne | 1 | 20* |  | 500* |  |  | 40* |  |  |  |  |  |
| Kiermusy | 3 |  | 113; 60;  (50-230) | 217; 4;  (0-610) | 3; 0; (0-10) |  |  |  |  |  |  |  |
| Bydgoszcz ZOO | 1 |  |  | 180* | 50* |  |  |  |  |  |  |  |
| Ustroń ZOO | 2 |  | 95; 95; (60-130) | 5; 5; (0-10) |  |  |  | 25; 25; (10-40) |  |  |  |  |
| Warsaw ZOO | 2 |  |  |  |  |  |  |  |  |  |  |  |
| Breeding centers | 14 |  |  |  |  |  |  |  |  |  |  |  |
| Pszczyna-Jankowice | 2 |  | 275; 275;  (40-510) | 1515; 1515; (1.280-1.750) | 125; 125; (110-140) |  | 5; 5; (0-10) |  | 50; 50; (0-90) | 20; 20; (0-40) | 25; 25;  (10-40) | 40; 40; (0-80) |
| Niepołomice | 3 |  | 460; 450; (350-580) | 1.170; 1.240; (80-2190) | 14; 10;  (0-310) |  |  |  | 20; 10; (0-50) |  |  | 30; 0; (0-80) |
| Smardzewice | 6 |  | 7; 5;  (0-20) | 148; 110; (10-510) | 7; 0; (0-30) |  | 3; 0; (0-10) |  | 3; 0; (0-20) |  |  |  |
| Wolisko | 3 |  | 100; 50;  (0-260) | 13.907; 12.240; (8.250-21.230) | 450; 370; (300-680) |  | 107; 10; (0-310) | 7; 0; (0-20) |  |  |  |  |
| Free-ranging | 7 |  |  |  |  |  |  |  |  |  |  |  |
| Białowieża Forest | 2 | 375; 375; (40-710) | 30; 30; (0-50) | 180; 180; (40-320) | 10; 10; (10) | 25; 25; (20-30) | 5; 5; (0-10) |  | 20; 20; (0-40) |  | 40; 40; (0-70) |  |
| Bieszczady Mountains | 3 | 13; 0 (0-40) | 430; 620; (0-680) | 477; 580; (50-800) |  |  | 67; 40; (0-160) |  |  |  | 33; 0; (0-100) | 7; 0; (0-20) |
| Borecka Forest | 1 |  |  |  |  |  |  |  |  |  |  |  |
| Knyszyn Forest | 1 | 90* | 80* |  |  |  |  |  |  |  |  |  |
| Total | 30 | 30; 0;  (0-710) | 141;  45; (0-680) | 1743; 130;  (0-21.230) | 70; 0;  (0-680) | 2; 0; (0-30) | 20; 0;  (0-310) | 2; 0;  (0-40) | 7; 0; (0-90) | 1; 0; (0-40) | 7; 0;  (0-100) | 6; 0; (0-80) |

*Only one examined European bison

Table S3. Effect of maintenance type, north gradient and sex of animals on nematode count for three nematode species. In the table only variables that were not excluded during selection of given model are present. In presented highest ranked models sex and north gradient were excluded for *O. ostertagi,* and maintenance type was excluded for *H. contortus* (B – beta coefficient, SE – standard error, Chi^2^ – Chi –squared test of significance, p – p value, 0* – reference category).

| Source | B | SE | Chi^2^ | p |
| --- | --- | --- | --- | --- |
| *O. ostertagi* | | | | |
| Intercept | 3.24 | 0.39 | 70.78 | <0.001 |
| Maintenance type (Enclosures) | -0.54 | 0.52 | 1.10 | 0.295 |
| Maintenance type (Breeding centers) | 2.62 | 0.47 | 31.18 | <0.001 |
| Maintenance type (Free ranging) | 0* |  |  |  |
| *O. lyrata* | | | | |
| Intercept | -16.60 | 8.31 | 4.00 | 0.046 |
| Maintenance type (Enclosures) | 0.61 | 0.99 | 0.38 | 0.539 |
| Maintenance type (Breeding centers) | 4.52 | 0.99 | 21.33 | <0.001 |
| Maintenance type (Free ranging) | 0* |  |  |  |
| Sex (Females) | -0.88 | 0.66 | 1.79 | 0.181 |
| Sex (Males) | 0* |  |  |  |
| North gradient | 0.30 | 0.16 | 3.60 | 0.058 |
| *H. contortus* | | | | |
| Intercept | 22.35 | 5.37 | 17.33 | <0.001 |
| Sex (Females) | 1.44 | 0.40 | 12.82 | <0.001 |
| Sex (Males) | 0* |  |  |  |
| North gradient | -0.41 | 0.11 | 14.80 | <0.001 |
